# PRimary Care Opioid Use Disorders treatment (PROUD) trial protocol: a pragmatic, cluster-randomized implementation trial in primary care for opioid use disorder treatment

**DOI:** 10.1186/s13722-021-00218-w

**Published:** 2021-01-31

**Authors:** Cynthia I. Campbell, Andrew J. Saxon, Denise M. Boudreau, Paige D. Wartko, Jennifer F. Bobb, Amy K. Lee, Abigail G. Matthews, Jennifer McCormack, David S. Liu, Megan Addis, Andrea Altschuler, Jeffrey H. Samet, Colleen T. LaBelle, Julia Arnsten, Ryan M. Caldeiro, Douglas T. Borst, Angela L. Stotts, Jordan M. Braciszewski, José Szapocznik, Gavin Bart, Robert P. Schwartz, Jennifer McNeely, Jane M. Liebschutz, Judith I. Tsui, Joseph O. Merrill, Joseph E. Glass, Gwen T. Lapham, Sean M. Murphy, Zoe M. Weinstein, Bobbi Jo H. Yarborough, Katharine A. Bradley

**Affiliations:** 1 Division of Research, Kaiser Permanente Northern California, 2000 Broadway, 3rd Floor, Oakland, CA 94612 USA; 2 Center of Excellence in Substance Addiction Treatment and Education, VA Puget Sound Health Care System, 1660 S Columbian Way, Seattle, WA 98108 USA; 3 Kaiser Permanente Washington Health Research Institute, 1730 Minor Avenue, Seattle, WA 98101 USA; 4 The Emmes Company, 401 N Washington St # 700, Rockville, MD 20850 USA; 5 Boston Medical Center/Boston University School of Medicine: Clinical Addiction Research & Education (CARE) Unit Crosstown Center, 801 Massachusetts Ave., 2nd Floor, Boston, MA 02118 USA; 6 Albert Einstein College of Medicine, Montefiore Medical Center, 3300 Kossuth Avenue, Bronx, NY 10467 USA; 7 Kaiser Permanente Washington, 9800 4th Ave. N.E., Seattle, WA 98115 USA; 8 Kootenai Clinic Family Medicine, 1919 Lincoln Way, Suite 315, Coeur d Alene, ID 83814 USA; 9 Department of Family & Community Medicine, McGovern Medical School, University of Texas Health Science Center at Houston School, 7000 Fannin Street, Houston, TX 77030 USA; 10 Department of Psychiatry, Center for Health Policy and Health Services Research, Henry Ford Health System, 2799 W Grand Blvd, Detroit, MI 48202 USA; 11 Department of Public Health Sciences, University of Miami Miller School of Medicine, 1120 NW 14th Street, 10th Floor, Miami, FL 33136 USA; 12 University of Minnesota/Hennepin Healthcare, 701 Park Avenue, Minneapolis, MN 55415 USA; 13 Friends Research Institute, 1040 Park Avenue, Suite 103, Baltimore, MD 21201 USA; 14 NYU Grossman School of Medicine, 180 Madison Ave., New York, NY 10016 USA; 15 Division of General Internal Medicine, Center for Research On Health Care, University of Pittsburgh School of Medicine, 200 Lothrop Street, 933West, Pittsburgh, PA 15213 USA; 16 University of Washington/Harborview Medical Center, 325 9th Ave, Seattle, WA 98104 USA; 17 National Institute on Drug Abuse Center for Clinical Trials Network, Three White Flint North, 11601 Landsdown Street, North Bethesda, MD 20852 USA; 18 Weill Cornell Medical College, 425 East 61st Street, Suite 301, New York, NY 10065 USA; 19 Clinical Addiction Research & Education (CARE) Unit, Boston University School of Medicine, Crosstown Center, 801 Massachusetts Ave., 2nd Floor, Boston, MA 02118 USA; 20 Kaiser Permanente Northwest, Center for Health Research, 3800 N. Interstate Avenue, Portland, OR 97227-1098 USA

**Keywords:** Medication, Buprenorphine, Opioid use disorder, Primary care, Nurse care manager, Collaborative care, Pragmatic trial

## Abstract

**Background:**

Most people with opioid use disorder (OUD) never receive treatment. Medication treatment of OUD in primary care is recommended as an approach to increase access to care. The PRimary Care Opioid Use Disorders treatment (PROUD) trial tests whether implementation of a collaborative care model (Massachusetts Model) using a nurse care manager (NCM) to support medication treatment of OUD in primary care increases OUD treatment and improves outcomes. Specifically, it tests whether implementation of collaborative care, compared to usual primary care, increases the number of days of medication for OUD (implementation objective) and reduces acute health care utilization (effectiveness objective). The protocol for the PROUD trial is presented here.

**Methods:**

PROUD is a hybrid type III cluster-randomized implementation trial in six health care systems. The intervention consists of three implementation strategies: salary for a full-time NCM, training and technical assistance for the NCM, and requiring that three primary care providers have DEA waivers to prescribe buprenorphine. Within each health system, two primary care clinics are randomized: one to the intervention and one to Usual Primary Care. The sample includes all patients age 16–90 who visited the randomized primary care clinics from 3 years before to 2 years after randomization (anticipated to be > 170,000). Quantitative data are derived from existing health system administrative data, electronic medical records, and/or health insurance claims (“electronic health records,” [EHRs]). Anonymous staff surveys, stakeholder debriefs, and observations from site visits, trainings and technical assistance provide qualitative data to assess barriers and facilitators to implementation. The outcome for the implementation objective (primary outcome) is a clinic-level measure of the number of patient days of medication treatment of OUD over the 2 years post-randomization. The patient-level outcome for the effectiveness objective (secondary outcome) is days of acute care utilization [e.g. urgent care, emergency department (ED) and/or hospitalizations] over 2 years post-randomization among patients with documented OUD prior to randomization.

**Discussion:**

The PROUD trial provides information for clinical leaders and policy makers regarding potential benefits for patients and health systems of a collaborative care model for management of OUD in primary care, tested in real-world diverse primary care settings.

*Trial registration* # NCT03407638 (February 28, 2018); CTN-0074 https://clinicaltrials.gov/ct2/show/NCT03407638?term=CTN-0074&draw=2&rank=1

## Introduction

Of the more than 2 million individuals in the United States with opioid use disorder (OUD), the vast majority do not receive treatment [1, 2]. Increasing treatment of OUD is critical, but not likely to be achieved unless it is provided in general medical settings in addition to specialty substance use treatment clinics [1, 3]. Buprenorphine and naltrexone are two FDA-approved medications with demonstrated efficacy for OUD, which can be provided in primary care (PC) settings [4, 5]. Although the use of medication treatment for OUD has increased over time, it reaches only 51% of privately insured patients with OUD [6], and merely 25% of Medicaid patients with OUD [7]. Most patients with OUD do not receive OUD treatment in PC [8, 9].

Substantial barriers to treating OUD in PC persist [10–14]. These include perceptions that treating OUD is out of the scope of PC practice, which lacks the time, resources, structure, and behavioral interventions required for OUD treatment [15]. Concerns are sometimes raised that PC cannot provide high quality OUD treatment [15]. Finally, many experts believe that pessimism and stigma pose major, if often unspoken, barriers [12]. Clinicians express concern that patients with OUD are difficult and could overwhelm PC practices [15].

Effective strategies exist to successfully implement high quality care for treating OUD in medical settings. A recent review identified 6 models of OUD treatment in PC [16]. Successful models generally relied on team-based approaches to address the above barriers. One of the models with the most support in the literature is the Massachusetts Collaborative Care Model [17–19]. The Massachusetts model (hereafter the “MA Model”) includes a full time OUD nurse care manager (NCM) as part of a team-based approach that shares care between the NCM and PC provider, with the nurse providing assessment, education, rapid access to medication, monitoring, and care coordination.

The MA Model has demonstrated effectiveness engaging and maintaining PC patients in OUD treatment, as well as attracting new patients into PC for OUD treatment [17, 18]. The model has been associated with high rates of persistent treatment at 12 months (51–67%) [17], and it is being tested in the recently funded and congressionally-mandated HEALing Communities multi-site trial [20]. However, the MA Model has been used predominantly in publicly-financed Federally Qualified Health Centers (FQHCs) in MA [17, 18, 21] and is not widely implemented outside MA. As implemented in FQHCs in MA [19], each NCM was expected to care for a panel of approximately 100 patients with OUD after full implementation, suggesting that the model could be cost-effective [18]. However, to date, other health systems have not made the upfront investment of hiring a full-time NCM, as required by the MA Model. This highlights the need for evidence of the MA Model’s feasibility, effectiveness and costs in other regions and across diverse health systems.

The PRimary care Opioid Use Disorder (PROUD) trial is testing whether implementing the MA Model in PC across six diverse health systems and regions can increase OUD medication treatment and secondarily improve outcomes of patients with OUD. The purpose of this report is to describe the PROUD protocol.

## Methods

### Objectives and hypotheses

The PROUD trial is a pragmatic, hybrid type III cluster-randomized implementation trial [22], a design which includes both a primary implementation objective and a secondary effectiveness outcome. PROUD tests strategies for implementing the MA Model in primary care, while secondarily evaluating the effectiveness of implementation for improving patient outcomes. The implementation objective (Objective 1; primary aim) is to test whether the MA Model, as compared to Usual PC in six diverse health systems, increases patient-days of medication treatment of OUD with either buprenorphine or extended release injectable naltrexone (XR-NTX) in PC, as documented in electronic medical records (EMRs) 2 years post-randomization. The primary implementation hypothesis is that the number of patient days of medication treatment of OUD is significantly greater in clinics randomized to the PROUD intervention compared to clinics randomized to Usual PC.

The effectiveness objective (Objective 2, powered secondary aim) evaluates whether the MA Model decreases acute care utilization in PC patients with OUD. The primary effectiveness hypothesis is that PC patients with documented OUD in the 3 years prior to randomization who receive care in PROUD intervention clinics, compared to those who receive care in Usual PC clinics, have fewer days of acute care utilization (e.g., in urgent care, emergency department [ED] and/or hospital) in the 2 years after randomization. This effectiveness objective reflects whether the PROUD intervention decreases acute health care utilization, a proxy for improved patient health outcomes.

Other study objectives include: (1) evaluating whether sex and race/ethnicity modify the impact of the PROUD intervention; (2) testing whether the intervention improves other implementation or effectiveness outcomes; and (3) identifying barriers and facilitators to implementation of the MA Model. In addition, since findings from the developmental phase of PROUD (Phase 1, described below) suggested that the recruited clinics may not be typical, observational analyses compare recruited clinics to other non-recruited (i.e. non-randomized) Usual PC clinics in the same system at baseline. Finally, four health systems in PROUD Phase 1 had unique care models for OUD treatment that health system leaders thought might be equal or superior to the MA Model. Thus, observational analyses compare outcomes in the PROUD intervention clinics to outcomes in these “exemplar” PC clinics.

### Overview of trial design

**Fig. 1:**
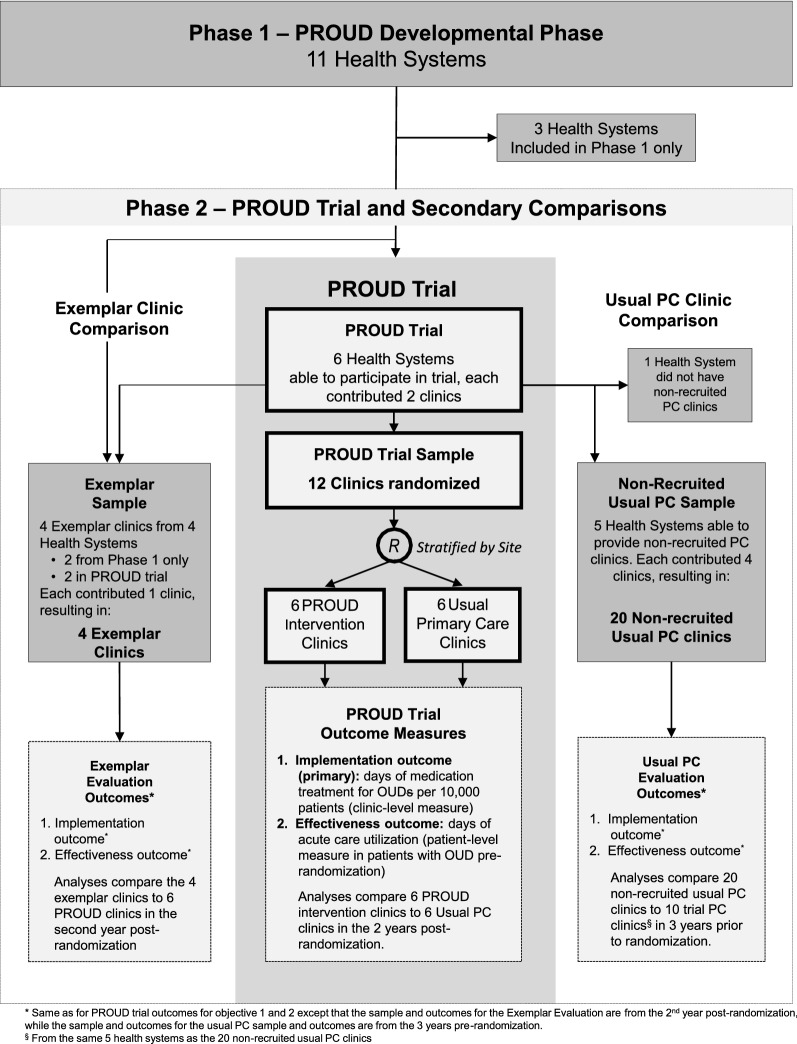
Schematic of PROUD study

The PROUD trial is being conducted in six health systems across the United States (Fig. 1). These health systems were recruited from among 11 health systems that participated in Phase 1. For PROUD Phase 2—the trial described in this report—each participating health system recruits two PC clinics willing to implement the MA Model, resulting in a total of 12 clinics across the six health systems. One of the two recruited PC clinics in each health system is randomly assigned to implement the MA Model, while the other is randomly assigned to continue Usual PC. The PROUD Data and Analytics Team obtains all quantitative data for sample identification and measures solely from existing electronic health records (EHRs), which include but are not limited to electronic administrative data, patients’ EMRs, and/or electronic data on health insurance claims, during a baseline (pre-randomization) period and follow-up (post-randomization) period. The PROUD Implementation Monitoring Team obtains qualitative data via: anonymous staff surveys at baseline and after the trial ends; stakeholder debriefs throughout the trial; and observations at site visits, trainings, and technical assistance (TA) throughout the trial. The PROUD trial leadership structure and teams are depicted in Additional file 1: Appendix S1; staff surveys and implementation monitoring tools are provided in Additional file 2: Appendix S2, Additional file 3: Appendix S3 and Additional file 4: Appendix S4. An independent, commercial Institutional Review Board (IRB) approved the study including providing waivers of consent and HIPAA authorization; all sites ceded to it.

### Conceptual framework

The PROUD Trial design is guided by the Practical, Robust Implementation and Sustainability Model (PRISM) framework for implementation, [23] which combines domains that impact the success of implementation. It includes four domains of barriers and facilitators to implementation: (1) the PROUD *intervention*, and how it interacts with recipients of the intervention; (2) the *recipients* of the intervention, including patient, clinician, and organization; (3) the implementation and sustainability *infrastructure* of the health system, including quality improvement teams, as well as space and EMR; and (4) the *external environment*, including regulatory policies, healthcare financing, and national quality measures [23]. These domains guide qualitative data collection by the PROUD Implementation Monitoring Team (See Data Sources). The PRISM’s outcome domains that reflect the success of implementation are contained within the RE-AIM framework: Reach, Effectiveness, Adoption, Implementation fidelity, and Maintenance of the intervention [24–26]. The first four RE-AIM domains guide selection of PROUD outcome measures.

### Developmental phase: PROUD Phase 1

The Developmental Phase of PROUD (Phase 1) lasted from January 2017 to November 2017. Objectives for Phase 1 were to select health systems that could obtain institutional support for the trial and demonstrate the feasibility of data collection. Phase 1 included 11 health systems from across the country that indicated they could provide the required data and possibly obtain permissions for participation in the main trial. From January to March 2017, lead investigators in the 11 Phase 1 health systems (“Site PIs”) worked to engage their health system leaders to assess whether the health system would be willing to participate in the PROUD trial. Six of the 11 Phase 1 sites were able to obtain the required support from all levels of their health systems within the required timeframe. Other activities during Phase 1 included preparation for data collection (e.g., development of measures and a data dictionary to create common data fields from the EMR and insurance claims data across the health systems) and obtaining approval of the NIDA National Drug Abuse Treatment Clinical Trials Network Data and Safety Monitoring Board. As above, one important finding of Phase 1 was that many clinics were unable to participate due to lack of leadership or PC provider support; consequently observational analyses were planned comparing recruited clinics to non-recruited (i.e. non-randomized) clinics in the same system at baseline. Another important finding of Phase 1 was that four health systems included PC clinics with unique models of OUD care (“exemplar” hereafter) that health system leaders felt were potentially as—or more—effective than the MA Model tested in the PROUD trial. As a result, observational analyses were also added to compare outcomes in the four exemplar clinics to outcomes in the six sites randomized to the PROUD intervention (Phase 2).

### Site selection for PROUD trial

Health systems were eligible for the PROUD trial based on: (1) health system and clinic leaders providing letters of support agreeing to participate in the trial, (2) having two adequately-sized PC clinics (i.e., approximately 10,000 unique patients with visits in a year) willing to participate by integrating a NCM into their clinic, (3) having at least three PC providers in the clinic willing to be waivered to prescribe buprenorphine, (4) a demonstrated ability to obtain the data necessary for the PROUD outcome measures—specifically days of treatment with medication for OUD and acute care utilization, (5) ability to meet all data sharing and regulatory requirements, (6) geographic, demographic and health system diversity, and (7) clinics not in close proximity to reduce cross-over potential. The six health systems selected included: two integrated insurance and delivery systems (Henry Ford Health System, MI; Kaiser Permanente [KP] Washington, WA), a community health system (Multicare Health System, WA), two university affiliated safety-net health systems (Harris Health System, TX; Montefiore Health System, NY), and a university-affiliated health system (University of Miami Health System, FL). For purposes of the trial, a “clinic” could be a cluster of two to three nearby smaller clinics that would function as a unit, sharing the NCM if randomized to the PROUD intervention. Three of the 12 recruited clinics are a cluster of two clinics (hereafter referred to simply as “clinics”).

### Phase 2: PROUD trial timeline

The PROUD trial includes three study periods: (1) startup; (2) intervention period (24 months); and (3) final data collection, analyses and dissemination (18 months).

- *Startup period* Start-up lasts from November 2017–February 2018 and includes arranging contracts and data use agreements with each health system, obtaining approval from a central IRB for all health systems, and preparing for randomization.
- *Intervention period* The 2-year intervention period (3/1/2018–2/29/2020) begins with randomization on 2/28/2018. The intervention period includes an estimated 6 months required to hire, onboard, and train a NCM, leaving about 18 months remaining for the NCM to support OUD treatment in the PROUD intervention clinic. During this period the Data and Analytics Team extracts limited datasets from the EMR and insurance claims data four times for reports to the Data and Safety Monitoring Board (with health systems deidentified) and refinement of data specifications and measures, while the Implementation Monitoring Team conducts both formative evaluation and qualitative data collection (described below).
- *Final data collection and analysis* Final trial data collection, cleaning, and analysis of the main and secondary trial outcomes occur in the 18 months (3/1/2020–8/31/2021) after the intervention period ends.

### Overview of the MA Model

The MA Model is a team-based, collaborative care approach that uses a full-time clinic-based NCM to integrate medication treatment of OUD into PC [17, 18]. The model is one of shared care between the NCM and the PC providers who prescribe OUD medications, in which agreed upon protocols, per the MA Model Office Based Addiction Treatment (OBAT) manual [27], allow the NCM to provide much of the routine care, with the provider’s role focused on diagnosis and treatment decisions, including referral to specialty addictions care when appropriate. The role of the NCM includes connecting with health system departments, hospitals, and community services so they can refer patients to PC for OUD treatment; assessment and support and engagement of patients seeking OUD treatment; coordinating with insurance plans; providing rapid access to intake assessments; coordinating prescriber visits to diagnose and prescribe buprenorphine or XR-NTX; support for medication initiation; monitoring and coordinating prescriptions refills for stable patients; checking the state Prescription Drug Monitoring Program; coordinating with outside agencies monitoring treatment; and monitoring urine drug tests. Although each health system chose how to use urine drug tests as part of care for OUD, in general, urine drug tests were used at the initial assessment of new patients and for monitoring during treatment to help guide clinical care. In the MA model, urine drug tests serve as a clinical tool in treating patients with OUD, allowing the NCM to have a conversation with patients and, when urine drug tests are positive or unexpected, allows the NCM to offer more support (e.g. increased frequency of visits), provide other tools or link to additional services, or talk about fentanyl, a “critical lab value,” which patients may not know was in their drug supply. The NCM role allows the PC provider to treat OUD in the normal flow of PC and minimizes additional workload. The NCM becomes an expert in OUD treatment and also plays an educational role to overcome barriers to OUD treatment both within the clinic and in the larger health system, with a focus on decreasing stigma by using non-stigmatizing language and normalizing OUD treatment in PC [17, 18, 28].

### The PROUD trial intervention

The PROUD trial intervention consists of three strategies to implement the MA Model: (1) providing funding and guidance to hire a NCM; (2) training and TA for the NCMs; and (3) PC provider training and mentoring. Further details of the three strategies are provided in Table 1. Formative evaluation [29] of implementation throughout the trial assesses whether barriers and/or facilitators necessitate refinements to implementation strategies.

**Table 1:** The PROUD intervention: three implementation strategies

(1) *Providing funding and guidance to hire a NCM* The PC clinic is provided funding for 1.0 Full Time Equivalent salary for a NCM for 2 years. The clinic leaders and health system then recruit, hire, and onboard a full-time NCM. Of note, although the PROUD trial provides financial support for the NCM salary and support for training and TA for the NCMs, health system leaders, not researchers, implement the MA Model in the intervention clinic. Further, the health system and its clinicians provide all clinical care to patients
(2) *Training and ongoing technical assistance (TA) *A TA team at Boston Medical Center provides training before the NCMs begin seeing patients and subsequent, ongoing support. This TA support includes training, an OBAT manual [27], a weekly videoconference to support and coach PROUD NCMs, and one-on-one consultation for questions as needed. The TA team’s OBAT nurses train the site NCMs in Boston for approximately 1.5–2 days. Training includes both didactic sessions and “shadowing” experienced NCMs while they provide care for patients with OUD. After each NCM is trained, a member of the TA team makes an in-person site visit to that intervention clinic offering training to all PC staff and meeting with the team providing PC OUD treatment. The weekly videoconferences consist of checking in with the NCMs about their patients, with a focus on problem-solving patient-, clinic- and system-level challenges, didactics on common challenges, and modeling of non-stigmatizing language and patient-centered care. A second site visit is optional to address challenges at the nursing level
(3) *PC providers trained and mentored* At least three PC providers are required to agree to prescribe buprenorphine, obtaining training and a Drug Enforcement Administration (DEA) waiver if not already waivered. Each PC prescriber is also asked to identify a mentor who can either be a local addictions expert in their health system or a mentor from a national program of voluntary mentors through the Providers Clinical Support System [30]. The TA team facilitates engagement between buprenorphine prescribers and their mentors during the site visit(s)

### PROUD comparison condition: usual primary care

Clinics randomized to Usual PC do not receive any resources or support from the study. Usual PC clinics are free to improve OUD treatment in any way they choose, but they are asked not to use the OBAT manual from Boston Medical Center to replicate the PROUD intervention in the Usual PC clinic. Usual PC is selected as the appropriate comparator because most PC clinics do not currently offer or have programs to support OUD treatment [16], but this could change as health systems respond to the opioid crisis.

### Primary care clinics used in observational comparisons

#### Non-recruited PC clinics

In order to assess whether the recruited (randomized) PROUD clinics differ from other PC clinics in the same system, all health systems in the PROUD trial were asked during Phase 1 if they could also provide the same EHR data needed for the trial, for four additional *non-recruited* PC clinics. Five health systems were able to provide such data; four eligible *non-recruited* PC clinics were selected for each of those 5 systems (Fig. 1).

#### Exemplar PC clinics

There are four exemplar clinics, two in systems participating in the PROUD trial (Montefiore and KP Washington) and two from other health systems from Phase 1 (KP Northwest and KP Colorado). In Montefiore and KP Washington, care for OUD had been implemented into routine care in one or more PC clinic(s), at a distance from the PROUD intervention clinics, with all or most PC providers prescribing buprenorphine; the largest in each system is chosen as the exemplar clinic. In KP Northwest and KP Colorado, a specialty addiction treatment program with features designed to lower barriers to OUD care, located in the same building or near a large PC clinic, is selected as the exemplar PC clinic (Fig. 1).

### Randomization

Randomization (1:1) is at the level of the PC clinic (n = 12), stratified by health system. As a result, six clinics (one clinic per health system) are assigned to the PROUD Intervention condition and six clinics (one clinic per health system) are assigned to the Usual PC condition.

### Ethical considerations

Waivers of informed consent and HIPAA Authorization are obtained for this study consistent with the three requirements of pre-2018 regulations (45 CFR 46.116(d)(3) [31]. These are appropriate because: (1) health system leaders and clinicians are implementing the MA Model in their system as part of improvements in clinical care; (2) risks are minimal when using secondary data with appropriate privacy safeguards, as in observational studies, without any contact between research staff and patients; and (3) the critically important questions about how to implement improved OUD treatment in PC could not practicably be answered without such waivers because recruiting patients and requiring provision of informed consent would result in a biased sample, compromising scientific validity of the assessment of the clinic-level intervention. A Data Safety Monitoring Board approved the protocol. All protocol modifications are approved by the IRB—and if important summarized on clinicaltrials.gov. The PROUD study has no interim stopping guidelines.

### Samples

#### PROUD trial

The PROUD sample includes PC patients age 16–90 years old with at least 1 in-person visit to a participating PC clinic (n = 12) from 3 years before to 2 years after randomization. The total sample of PC patients in the trial is anticipated to be over 170,000 across the 12 clinics in the six health systems. The main implementation objective (primary outcome of days of OUD treatment) is assessed in the full study sample of all PC patients with visits during the intervention period (from randomization to trial end). The main effectiveness objective (days of acute care utilization) is evaluated in the subsample of PC patients who have documentation of an OUD diagnosis in their EHR data up to 3 years prior to randomization.

Samples for observational analyses of non-recruited Usual PC clinics and exemplar clinics parallel the main trial sample inclusion criteria for implementation or effectiveness outcomes.

### Data sources and collection

#### Quantitative data sources available for all PC clinics in the trial

All quantitative measures for randomized, non-recruited Usual PC, and exemplar clinics are secondary data ascertained from EHRs—health system administrative databases, EMRs, and insurance claims—with insurance claims only available from two sites that provide health insurance for some of their patients. Data domains include demographics, diagnoses, outpatient medication orders for medications of interest [(OUD treatments, naloxone, opioids, sedative hypnotics, stimulants, antidepressants, muscle relaxants, and medications to treat human immunodeficiency virus (HIV), Hepatitis C virus (HCV), alcohol use disorder (AUD), and neuropathy)], pharmacy dispensing data (if available), procedures, select laboratory tests including urine drug tests and HCV and HIV related laboratory tests, health care utilization, and deaths documented in EHRs. Final data collection occurs 7 months after the end of the trial, allowing 6 months for the lag in insurance claims data (two health systems). The Data Safety Monitoring Board reviews data collected, including safety data (e.g. overdose) approximately every 6 months. Data quality checks and information on data management, storage and security can be obtained from the lead investigator. If the intervention increases OUD treatment, funding for an ancillary study of the impact on mortality will be sought, including data from the National Death Index (NDI).

#### Data available only from PROUD intervention clinics

The NCM provides weekly counts of patients to the TA team as part of ongoing weekly support, and these data are also provided to the Implementation Monitoring Team. Each week the NCM sends counts of the following: the number of new patients the NCM talked to about treatment; the number of full OUD intake assessments; the number of patients who newly started treatment, were re-engaged, or who transferred OUD care to the intervention clinic (i.e. had already started medication treatment for OUD elsewhere); the number of scheduled follow-up and walk-in patients seen; the number of XR-NTX injections given; the number of no shows for scheduled NCM visits, and the number “discharged” (screened but never started on medication, incarcerated, transferred to higher level of care, lost to follow-up, deceased, or discharged due to administrative or medical reasons). From these data, weekly summary counts of total number of patients ever managed by the NCM and total number of patients in treatment currently at each intervention clinic are shared with Site PIs, the Implementation Monitoring Team, and trial leadership.

#### Anonymous PC staff survey

An anonymous survey of PC staff in PROUD intervention and Usual PC clinics is conducted at baseline and after the end of the trial. This survey asks each staff member for their role and years in practice (generally and in the clinic), and includes eight questions about the appropriateness of, feasibility of, and attitudes toward treating OUD in their PC clinic (Appendix S3).

#### Qualitative data source: ongoing implementation monitoring

An Implementation Monitoring Team holds weekly meetings to identify barriers and facilitators to implementation across four domains: intervention; recipients (patients, clinicians, staff, and leaders); healthcare infrastructure; and the external environment [23] to inform any adaptations to the intervention strategies to enhance implementation (i.e., formative evaluation) and collect contextual data to support interpretation of trial results. The Implementation Monitoring Team includes four study team members from the lead investigative team, including the lead investigator.

##### Qualitative data from all PROUD clinics

To understand the full context of OUD care at each clinic (intervention and control), the Site PI(s) and project manager at each of the six health systems conduct formal interviews with key informants in their health systems at baseline and then every 6 months (Appendix S2; Appendix S4). These interviews cover four qualitative domains of the PRISM [23] model—(1) the intervention itself, (2) recipients of the intervention including the health system—organizational characteristics, leaders, managers, staff and patients, (3), implementation and sustainability infrastructure and (4) external environment [23]. Once the site PI(s) and project manager in each health system have completed their key informant interviews, the Implementation Monitoring Team debriefs the Site PI(s) and project manager on what they learned from their interviews with regard to potential barriers and facilitators to OUD treatment and implementation, and monitor changes over time related to the context of implementation and care for patients with OUD in each health system. Parallel assessments of OUD care and other contextual factors are also conducted for exemplar clinics.

##### Qualitative data on intervention clinics only

The Implementation Monitoring Team also collects qualitative data specific to the intervention clinics to understand the implementation and its recipients, as well as for formative evaluation. Data sources include: *observations* of NCM trainings in Boston, the TA team’s site visit(s) to intervention clinics, and weekly TA team videoconference calls with the NCMs; bi-monthly to monthly debriefs with the TA team to review facilitators and barriers from all sites; *review of email communications* using a central study email box for all trial-related communication, *site debriefs* with Site PIs and project managers including periodic “all-site” phone meetings (initially weekly and decreasing frequency over the trial) and ad hoc or scheduled debriefs with a single site. These qualitative data are used as part of formative evaluation to provide feedback at weekly leadership meetings leading to discussions about whether refinements or adaptations of the three implementation strategies are needed.

### Measures

#### Objective 1: implementation outcome—patient days of OUD medication treatment

The number of patient days of medication treatment of OUD documented in the EMR in each clinic in the 2 years post-randomization is the primary outcome (Table 2). To account for varying clinic sizes, the outcome is divided by the number of patients seen in the clinics during that time period and then multiplied by an appropriate scaling factor in order to report the results (e.g., multiplying by 10,000 to calculate the number of patient days of OUD treatment per 10,000 patients), and reported as patient-years of treatment provided by a clinic (calculated by simply dividing days of OUD treatment by 365). This measure was selected as the primary outcome because: it reflects both initiation and retention and thus both access and quality; it is continuous and therefore maximizes statistical power in a study with a small number of clusters; and it can be estimated in the entire sample of PC patients to avoid identification bias [32].

**Table 2:** Primary, secondary, and other outcomes measured during the 2 years post-randomization based on EHR data, as entered into clinicaltrials.gov

Outcome measures
* Objective 1. Patient-days of OUD medication treatment (primary outcome)* Clinic-level number of patient-days of OUD treatment with buprenorphine and XR-NTX documented in the EHR during the period from randomization until 2 years after, reported per 10,000 PC patients in the clinic in the 2 years post-randomization
* Objective 2. Acute care utilization (secondary outcome)* Patient-level number of days of acute care utilization during the period from randomization until 2 years after, among patients with an OUD diagnosis documented in the EHR in the 3 years prior to randomization
Other outcome measures of implementation
* Newly diagnosed OUD (implementation reach)* Clinic-level number of patients with a new International Classification of Disease (ICD) code for OUD documented in the EHR during the period from randomization until 2 years after who did not have an OUD diagnosis documented in the EHR in the 3 years prior to randomization, reported per 10,000 patients in the PC clinic in the 2 years post-randomization
* Initiation*^*a*^* of OUD treatment (implementation reach)* Clinic-level number of patients who initiate: (1) buprenorphine or (2) XR-NTX with an OUD diagnosis as documented in the EHR during the period from randomization until 2 years after, reported per 10,000 PC patients in the clinic in the 2 years post-randomization
* Retention in OUD treatment (implementation fidelity)* Clinic-level number of patients initiating^a^ OUD treatment during the period from randomization until 2 years after randomization as documented in the EHR, who also receive OUD treatment on 80% of days available after initiation reported per 10,000 PC patients in the clinic in the 2 years post-randomization [34]
* Naloxone prescribing (implementation fidelity)* Patient-level number of prescriptions of naloxone for overdose management in the period from randomization until 2 years after, among patients with an OUD diagnosis in the 3 years prior to randomization
Other outcome measures of effectiveness
* Urgent care or ED use* Patient-level number of visits to urgent care or EDs during the period from randomization until 2 years after, among patients with an OUD diagnosis documented in the EHR in the 3 years prior to randomization
* Inpatient days hospitalized* Patient-level number of days hospitalized during the period from randomization until 2 years after, among patients with an OUD diagnosis documented in the EHR in the 3 years prior to randomization

^a^Initiation of buprenorphine and XR-NTX in the context of PROUD trial outcome measures refers to an order for OUD medication treatment post-randomization with no treatment with these medications in the prior 365 days

Medication treatment for OUD includes buprenorphine formulations that are FDA approved for OUD or XR-NTX; methadone is not included since data from methadone opioid treatment programs (OTPs) are not available in EMRs and most of the health systems do not have data on methadone OTPs. An OUD diagnosis for buprenorphine is not required because Phase 1 analyses revealed OUD diagnoses are sometimes missing, consistent with the literature [7], and an OUD diagnosis is more likely to be documented for patients in Intervention compared to Usual PC clinics, so that requiring an OUD diagnosis could bias findings toward favoring the intervention. Since XR-NTX is also FDA-approved for AUD, when both OUD and AUD are documented, XR-NTX is considered OUD treatment if the number of documented OUD diagnoses are about equal to AUD (i.e. within two). Medication orders from EMRs are the basis for the primary outcome. This outcome measure was selected because not all sites had data on medication dispensing from pharmacies or insurance claims for contracted OUD treatment in their EHRs, and the trial requires an outcome that is comparable across all sites. However, pharmacy dispensing data and insurance claims and other EHR data, when available, are used for secondary measures.

#### Objective 2: main effectiveness outcome—days of acute care utilization

Acute care utilization, a count measure of the number of days of acute care utilization in the 2 years after randomization, is the secondary outcome. It is the sum total of visits to urgent care clinics (not same day appointments in PC) and emergency departments (EDs), and days hospitalized. This outcome was selected because ED and hospital care are widely available proxy measures for adverse outcomes of OUD, hypothesized to improve in patients with OUD who receive timely medication treatment, and less susceptible to documentation biases than specific diagnoses (e.g., overdose).

#### Other outcomes

Other pre-specified outcomes for implementation and effectiveness based on EHR data are shown in Table 2 [33]. Additional exploratory and explanatory measures, planned both a priori or during the trial prior to data cleaning and locking of the database, are outlined in the final Statistical Analysis Plan (available from authors).

### Analyses

#### Qualitative analyses

Implementation-focused formative evaluation [29] is conducted throughout the trial to assess whether the three implementation strategies—salary for a NCM, TA and three PC providers obtaining training and DEA waivers for buprenorphine treatment—needed any refinement. Formative evaluation uses qualitative data on observed barriers and facilitators from each site, and iterative discussions among trial leaders, to arrive at any decisions about refinement of implementation strategies.

To provide context for interpretation of quantitative findings, the Implementation Monitoring Team also identifies barriers and facilitators to implementation using a rapid coding process [35] as in prior studies [36]. This coding process consists of four steps. First, at the end of the study the Implementation Monitoring Team members each identify up to 10 important barriers and facilitators of implementation for each health system. Second, after discussion, the Implementation Monitoring Team arrives at a consensus regarding key barriers and facilitators for each site. Third, results for each site are shared with stakeholders (the NCM, Site PIs and Site project managers, and the TA team), for feedback on appropriate representation of context and revised accordingly. Finally, barriers and facilitators are categorized into the PRISM’s four domains described above: (1) intervention; (2) recipients—patients, clinicians, managers, staff and organizational leaders; (3) health system and clinic implementation infrastructure; and (4) external environment [23].

### Statistical analysis

#### Objective 1: main implementation objective (primary aim)

Main analyses are based on intent-to-treat (“per randomization”), and compare PROUD intervention and Usual PC clinics regarding the clinic-level number of patient-days of medication treatment of OUD (per 10,000 patients seen) over the 2 years after randomization. Analyses fit a mixed-effect model to account for correlation of outcomes from the pair of clinics from the same health care system,

$${y}_{ij}=\alpha +\beta *tr{t}_{ij}+{\gamma *{z}_{ij}+\theta }_{j}+{\epsilon }_{ij}$$

where ${y}_{ij}$ is the primary outcome measure for clinic $i$ at health care system $j$, $tr{t}_{ij}$ is the treatment indicator (for PROUD intervention versus Usual PC), and ${z}_{ij}$ is the observed “baseline” value of the outcome defined over the 2 years prior to randomization. Additionally, ${\theta }_{j}$ is the random effect for health system $j$, assumed to be normally distributed with a common variance, and ${\epsilon }_{ij}$ is the error term (also assumed to be normally distributed). To evaluate whether the PROUD intervention increases EMR-documented OUD treatment, analyses test whether $\beta$ significantly exceeds zero using a one-sided hypothesis test at the 0.05 level. This is appropriate because our primary aim is to test superiority of implementation of the MA model relative to Usual PC in order to inform health systems’ decisions as to whether to implement this model of OUD care.

Given the small number of clusters and the potential for chance imbalance in covariates, secondary analyses are conducted adjusting for covariates predictive of OUD medication treatment days during the baseline period; these are identified using baseline data to select at most two covariates most strongly associated with days of OUD medication treatment during the baseline period (given the limited available degrees of freedom). Other secondary analyses of the primary (implementation) outcome include: site-specific (descriptive) analyses comparing PROUD intervention and Usual PC clinics at each site; “per protocol” analyses (1) restricted to health systems whose intervention clinic’s NCM treated over 30 patients (indicating successful implementation), or (2) using a modified definition of the follow-up period over which the Objective 1 outcome measure is calculated (e.g., after the NCM began seeing patients to account for delay in implementation due to NCM hiring and training). Additional sensitivity analyses are conducted in which the outcome specifications are varied (e.g., using the most complete EHR data available from each site by including pharmacy dispensing and insurance claims data from sites where it is available). Descriptive analyses also evaluate crossover from a Usual PC clinic pre-randomization to the PROUD intervention clinic post-randomization in patients with OUD. Analyses of other pre-specified implementation outcomes (Table 2) use largely the same general approach as for the primary outcome. Exploratory outcomes also describe different dimensions of implementation based on the PRISM model such as adoption (e.g., proportion of PC providers who prescribe buprenorphine), implementation fidelity (e.g., use of urine drug tests). Differences between intervention and Usual PC clinics, and any changes in PC staff attitudes from baseline to after the trial ends will also be assessed.

#### Objective 2: main effectiveness objective

Analyses to assess effectiveness evaluate, among individuals who have an OUD diagnosis prior to randomization, whether acute care utilization during follow-up differs between those in clinics assigned to the PROUD intervention and those in Usual PC clinics. The primary analyses of effectiveness exclude patients newly diagnosed with OUD post-randomization, as these patients could differ systematically between PROUD intervention and Usual PC clinics, if the MA Model attracts new patients into PC [18], which could lead to biased estimates of the treatment effect [32]. A mixed-effect Poisson regression model (with log link) is fit at the patient level to the number of days of acute care utilization. The model adjusts for the baseline value of the outcome (count measure of the number of days of acute care utilization) and accounts for clustering of patients within a clinic by including clinic-specific random intercepts. If any site randomizes after 2/28/2018, analyses would also adjust for time from randomization to the end of the trial (2/29/2020) by including an offset term. The Objective 2 effectiveness hypothesis is evaluated by testing whether the coefficient of the intervention group assignment (PROUD versus Usual PC) differs from zero using a two-sided test at the 0.05 level with a small-sample correction method given the small number of clusters in the analysis [37, 38].

Several secondary effectiveness analyses of the Objective 2 outcome are planned. Examples include adjusting for additional patient-level variables and expanding the analytic sample to include patients with newly documented OUD post-randomization. In addition, other effectiveness outcomes (Table 2) are analyzed using a similar approach as for the Objective 2 outcome*.*

### Differences in PROUD intervention impact on Objective 1 outcome across age, sex and race/ethnicity

Given the importance of understanding how the MA Model improves care for subpopulations, subgroup analyses are conducted based on: age (< 26 vs. older); sex; race and ethnicity. Any such comparisons are likely underpowered and must be interpreted with caution. The original Massachusetts studies observed that patients who were male or Black/African American or Hispanic were less likely to be retained in PC treatment of OUD with the MA Model, compared to female and white patients, respectively [17, 28], but no differences were observed across age groups. As a result, PROUD investigators hypothesize that the intervention will result in smaller increases in OUD medication treatment in patients who are male or Black/African American or Hispanic, but hypothesize no differences across age groups.

### Planned observational comparisons

#### Observational comparisons

Several observational comparisons are planned. To evaluate whether PC clinics in the PROUD trial differed from other PC clinics in the same health systems, recruited and non-recruited PC clinics are compared in the five health systems able to provide data on four non-recruited (non-randomized) Usual PC clinics. Specifically, patients in the 10 recruited clinics are compared to 20 non-recruited PC clinics during the pre-randomization period in these five health systems (Fig. 1). Implementation and effectiveness outcomes in the second year after randomization are also compared in observational analyses between the six intervention clinics and the four exemplar programs for OUD, described above.

#### Economic analyses

The PROUD trial is providing data for an ancillary economic analysis of the health system costs with the following outcomes: (a) the cost of implementing the MA Model; (b) the mean per-person daily cost of delivering the MA Model on an ongoing basis; (c) the differences in the mean per-person costs associated with PC, behavioral healthcare, ED, and inpatient services between the study arms over the post-randomization observation period; (d) the difference in the average total cost of healthcare service utilization between the study arms over the post-randomization observation period; and (e) the difference between (d) and (b), which represents the incremental net benefit of the PROUD intervention. A description of the protocol for this ancillary study will be forthcoming in a separate publication.

### Power

#### Objective 1 (primary aim)

Power calculations for Objective 1 assessed whether there is sufficient power (> 80%) to detect a fivefold increase in the number of patient days of OUD medication treatment associated with the PROUD intervention as compared to Usual PC. There is no agreed upon benchmark for high quality OUD treatment, and an increase of this magnitude, which could reflect a fivefold increase in the number of patients who access and/or are retained in treatment, was felt to be clinically-relevant and a substantial enough increase to convince policy-makers. Simulations, using PROUD phase 1 data, were conducted to calculate power under the planned analytic approach, as described above (mixed-effect regression model that adjusts for the baseline value of the outcome), with a one-sided test with a type 1 error rate of 0.05. Parameter values for the simulation were estimated using Phase 1 data on the 12 clinics recruited into the PROUD trial (two clinics from each health system). Details on the data-generating model for the simulation along with the specific parameter values are in the study Statistical Analysis Plan (available from authors). Based on these simulations, the PROUD trial is estimated to have at least 80% power to detect a 30% increase in the number of OUD-treated days per patient seen in PC. Because 30% is far smaller than the fivefold (400%) clinically- or policy-meaningful increase, the study is sufficiently powered with two clinics in each of six health care systems to detect the targeted fivefold increase in the primary outcome measure.

#### Objective 2 (secondary aim)

Power calculations for Objective 2 used simulations to calculate the power to detect a reduction in acute care utilization among patients with an OUD diagnosis pre-randomization, comparing PROUD intervention versus Usual PC clinics, under a range of assumed values of the effect size, as well as a range of assumed values for the proportion of patients with OUD treated by the PROUD NCM. Outcome data on acute care utilization for simulations were generated using a Poisson mixed-effect model with sample size and parameters estimated using Phase 1 data for the 12 clinics recruited into the PROUD trial. These simulations assumed that among patients with OUD in the PROUD intervention clinic, those who visit the PROUD NCM and receive sustained treatment with buprenorphine or XR-NTX would have 10–20% of the rate of acute care utilization (i.e., a relative rate $R{R}_{treat}$ of 0.1–0.20) as patients with OUD who are not treated for OUD by the PROUD NCM, since sustained treatment for OUD is hypothesized to markedly reduce acute care utilization [39]. To each simulated dataset a Poisson mixed-effect model was fit applying the between-within (BW) DF corrected F test [37] with a (2-sided) type-1 error rate of 0.05. Under effect size of $R{R}_{treat}$ of 0.1–0.2 the PROUD trial has over 80% power if at least 39–44% of patients with OUD in the PROUD intervention arm pre-randomization are treated for OUD.

### Trial status

The PROUD Trial intervention ends 2/29/2020. Final quantitative and qualitative data collection occurs March–September 2020. Main results are expected by 8/2021. Results will be published in peer reviewed journals.

## Discussion

Despite the availability of first-line medication treatment for OUD that can be provided in PC, many practices do not offer such treatment. The PROUD trial seeks to address this gap by implementing a promising model, the MA model, into PC to engage patients in OUD treatment in medical settings. The MA Model is a collaborative care model designed to support treatment of OUD in PC practices by providing nurse support for all elements of care, except OUD diagnosis and medication prescribing [17, 18, 28]. This model of shared-care between a PC provider and NCM was also designed to address knowledge gaps, lack of time, need for clinic support, and stigma, which are common barriers to treatment of OUD in PC [11, 12, 15]. Promising reports [17–19] suggest that the MA Model allows a nurse to develop deep expertise in OUD treatment, supported by national experts, as a way to improve access, overcome stigma and support the development of local PC expertise in OUD treatment [17–19]. The model also allows PC providers to treat many more patients with OUD than they could alone—up to 100 per NCM [18]. The PROUD trial is designed to provide information for clinical leaders and policy makers regarding benefits and costs of the MA Model by testing the model in real-world PC settings in diverse health systems. The trial relies on health systems to implement the model and uses secondary data to identify the study samples and evaluate implementation and effectiveness outcomes. As a result, all PC patients who visited the randomized clinics over the study period are included in primary analyses. The PROUD trial can provide a roadmap for other health systems searching for a practical care model for OUD treatment in PC.

### Limitations and strengths

In this pragmatic trial that relies on EHR data for defining the primary and secondary outcomes, the data can be a limitation to the trial’s internal validity. For the primary outcome (days of medication treatment for OUD), orders for buprenorphine or XR-NTX in the EMR could be written and never dispensed or ingested (for oral medications), and patients could obtain OUD treatment outside the participating health systems - including methadone from opioid treatment programs—that may not be documented in the EHR [40]. There is no way to determine whether services were not provided or whether the data are missing, and external OUD treatment data are not included in the primary outcome measure because they are unavailable at most sites. However, randomization is stratified within health system and the participating health systems provide the vast majority of care for the populations they serve; therefore, limitations in outcome data should be similar in both arms within a site. Limitations of data sources are also addressed with sensitivity analyses which vary the specification of the outcome (e.g., to include dispensing of medications, insurance claims, and methadone data at sites where available) to estimate whether this meaningfully changes the main results. Incomplete ascertainment of acute care utilization could also under-estimate or bias results regarding effectiveness (Objective 2) as only two participating systems are health insurance plans with claims data. However, analyses of acute care utilization among patients with OUD are adjusted for patients’ pre-randomization acute care utilization, and stratified randomization ensures that systematic differences in ascertainment between health systems are balanced across intervention groups. Another limitation is that only some adverse health effects of OUD lead to urgent, emergency, or hospital care, and although other outcomes are evaluated, many adverse outcomes of OUD cannot be ascertained from EHRs. Further, many PC patients with OUD do not have a documented OUD diagnosis and are therefore excluded from Objective 2 analyses, and others may leave or obtain care outside the health system, which can only be ascertained for the two systems with health insurance plans and claims data. However, sensitivity analyses are planned that include patients with documented OUD post-randomization or require PC visits post-randomization (as a proxy for continued enrollment). In addition, if the trial finds that the MA Model meaningfully increases OUD treatment, a future planned ancillary study will obtain population-based death data from the National Death Index to evaluate the intervention’s impact on death among patients with documented OUD.

Limitations to external validity relate to the cost of the nurse, which is not borne by the health systems, and the fact that sites recruited are a select group of clinics with a leader and/or clinicians willing to participate in the trial. The MA Model’s requirement for a large upfront investment in a full-time NCM and regional shortages of nurses might limit generalizability. However, additional observational comparisons evaluate outcomes in the PROUD intervention clinics compared to potentially more generalizable “exemplar” models of OUD care already in place and compare recruited and randomized PC clinics to non-recruited PC clinics before randomization. Prior research suggests that some PC providers are not willing to prescribe, and models that utilize non-physicians may be needed [4]. Finally, a limitation of using “usual care” as a comparison is that its characteristics must be clearly described at baseline and changes over the course of a trial must be clearly ascertained and described [41]. To understand usual care provided in control clinics (and its generalizability), the Implementation Monitoring Team conducts robust assessment at baseline and monitoring throughout the study, with qualitative and quantitative data collected by each site’s PI and project manager, while avoiding contact between researchers and sites.

At the same time, the PROUD trial has important strengths related to its design, sample, and innovative measures allowing evaluation of the MA model in diverse naturalistic settings. The pragmatic design of the trial allows the study of a sample of patients with OUD seen in PC who seldom seek OUD treatment or enroll in trials. Further, the design allows evaluation of treatment engagement and acute care outcomes over a 2-year follow-up period. Each system agreed to implement the MA Model in one randomly selected PC clinic and to provide data from 3 years before and 2 years after randomization. The trial obtains adequate statistical power for a trial with 12 clinics by using a novel primary outcome measure of patient-days of medication treatment for OUD per 10,000 PC patients seen, which reflects both access and retention. The trial design also allows evaluation of the extent to which the MA Model attracts new patients into PC for OUD treatment or leads to newly recognized OUD in the intervention clinics. An innovative Implementation Monitoring Team assesses usual care at baseline and monitors usual care and intervention implementation over time, identifying barriers and facilitators in each system, which allows interpretation of variation in outcomes across sites. Further, the trial includes geographically diverse health systems that serve racially and ethnically diverse populations and have varying organization of health care delivery and financing, including two safety net systems, one academic system, one regional healthcare delivery system, and two systems that integrate health insurance with care delivery.

## Conclusion

The PROUD trial is an innovative pragmatic trial that is evaluating the ability of the MA Model to improve access to and retention in OUD treatment in PC clinics in six diverse health systems. Results will provide critical evidence to help health system leaders evaluate whether investing upfront in a full-time NCM for OUD treatment increases OUD treatment in PC and decreases the need for acute care.

## Supplementary Information

**Additional file 1: Appendix S1.** Organization of the PROUD Study Investigative Teams.

**Additional file 2: Appendix S2.** Baseline PROUD implementation monitoring information.

**Additional file 3: Appendix S3.** Clinic staff survey.

**Additional file 4: Appendix S4.** Quarterly PROUD debrief between Site PI/PM and PROUD Implementation Monitoring Team.

## Data Availability

The protocol, the statistical analysis plan and code are available from the lead author upon written request. The final dataset will be analyzed by investigators at the Emmes Company and can be made available as de-identified data to investigators upon reasonable request with the required IRB approval and data use agreements.

## Abbreviations

AUD: Alcohol use disorder
DEA: Drug Enforcement Administration
ED: Emergency Department
EHR: Electronic health record
EMR: Electronic medical record
FDA: Federal Drug Administration
FQHC: Federally Qualified Health Center
HCV: Hepatitis C virus
HIV: Human immunodeficiency virus
ICD: International Classification of Disease
KP: Kaiser Permanente
MA Model: Massachusetts Model
NCM: Nurse care manager
NDI: National Death Index
NIDA: National Institute on Drug Abuse
OBAT: Office based addiction treatment
OTP: Opioid treatment program
OUD: Opioid use disorder
PC: Primary care
PRISM: Practical, Robust Implementation and Sustainability Model
PROUD: PRimary Care Opioid Use Disorders treatment (PROUD)
RE-AIM: RE-AIM: Reach, Effectiveness, Adoption, Implementation fidelity, and Maintenance
TA: Technical assistance
XR-NTX: Injectable naltrexone
