# Supplementary material for: PRimary Care Opioid Use Disorders treatment (PROUD) trial protocol: a pragmatic, cluster-randomized implementation trial in primary care for opioid use disorder treatment
Source: Addict Sci Clin Pract. 2021 Jan 31;16:9. doi: 10.1186/s13722-021-00218-w (PMC7849121; doi:10.1186/s13722-021-00218-w)
Supplement: Supplementary file 1 — Additional file 1: Appendix S1. Organization of the PROUD Study Investigative Teams. [file 13722_2021_218_MOESM1_ESM.pptx]

## Slide 1
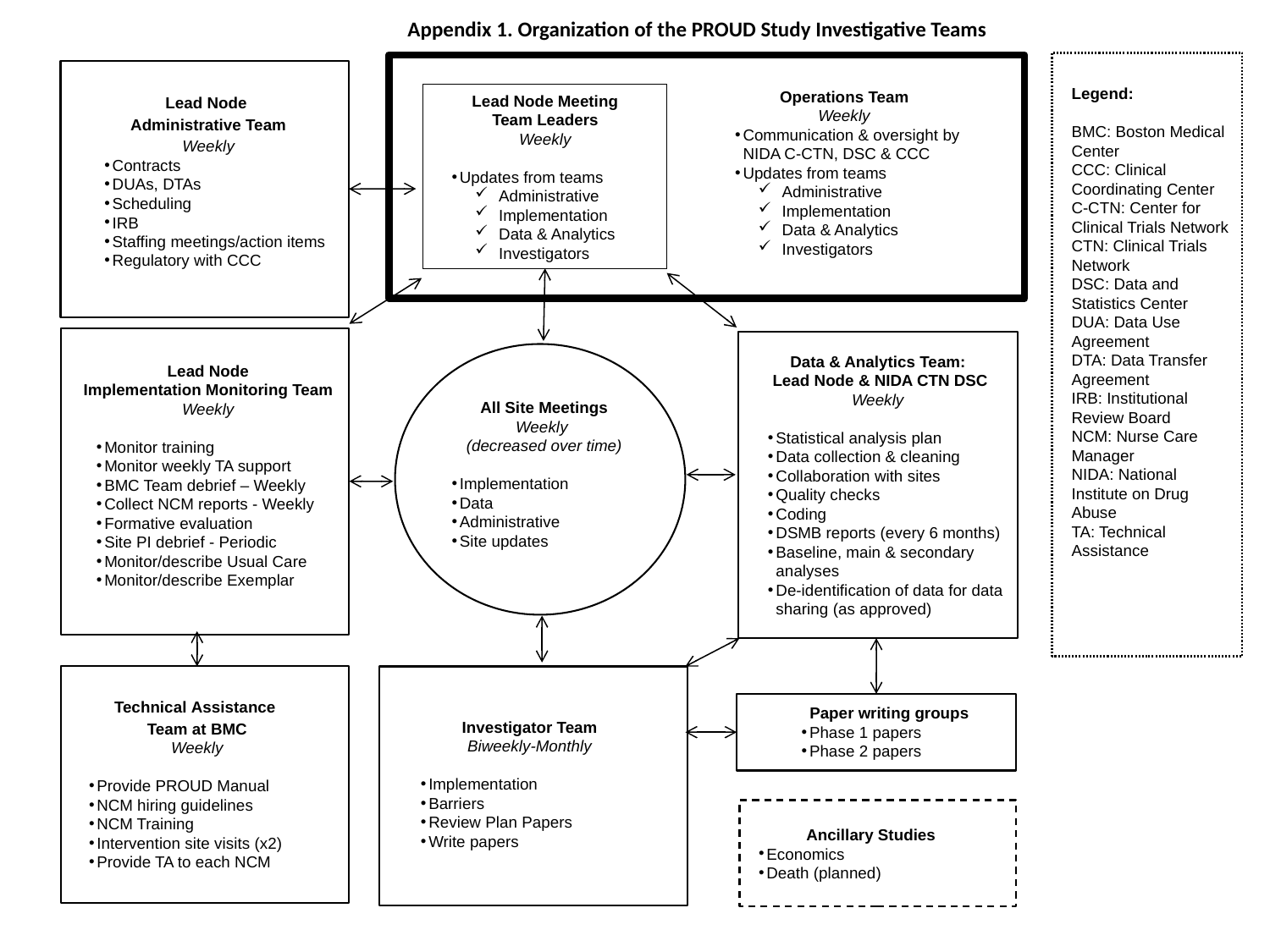

Appendix 1. Organization of the PROUD Study Investigative Teams
Operations Team
Weekly
Communication & oversight by NIDA C-CTN, DSC & CCC
Updates from teams
Administrative
Implementation
Data & Analytics
Investigators
Legend:
BMC: Boston Medical Center
CCC: Clinical Coordinating Center
C-CTN: Center for Clinical Trials Network
CTN: Clinical Trials Network
DSC: Data and Statistics Center
DUA: Data Use Agreement
DTA: Data Transfer Agreement
IRB: Institutional Review Board
NCM: Nurse Care Manager
NIDA: National Institute on Drug Abuse
TA: Technical Assistance
Lead Node
Administrative Team
Weekly
Contracts
DUAs, DTAs
Scheduling
IRB
Staffing meetings/action items
Regulatory with CCC
Lead Node Meeting
Team Leaders
Weekly
Updates from teams
Administrative
Implementation
Data & Analytics
Investigators
Lead Node
Implementation Monitoring Team
Weekly
Monitor training
Monitor weekly TA support
BMC Team debrief – Weekly
Collect NCM reports - Weekly
Formative evaluation
Site PI debrief - Periodic
Monitor/describe Usual Care
Monitor/describe Exemplar
Data & Analytics Team:
 Lead Node & NIDA CTN DSC
Weekly
Statistical analysis plan
Data collection & cleaning
Collaboration with sites
Quality checks
Coding
DSMB reports (every 6 months)
Baseline, main & secondary analyses
De-identification of data for data sharing (as approved)
All Site Meetings
Weekly
(decreased over time)
Implementation
Data
Administrative
Site updates
Technical Assistance
Team at BMC
Weekly
Provide PROUD Manual
NCM hiring guidelines
NCM Training
Intervention site visits (x2)
Provide TA to each NCM
Paper writing groups
Phase 1 papers
Phase 2 papers
Investigator Team
Biweekly-Monthly
Implementation
Barriers
Review Plan Papers
Write papers
Ancillary Studies
Economics
Death (planned)
