# Supplementary material for: PRimary Care Opioid Use Disorders treatment (PROUD) trial protocol: a pragmatic, cluster-randomized implementation trial in primary care for opioid use disorder treatment
Source: Addict Sci Clin Pract. 2021 Jan 31;16:9. doi: 10.1186/s13722-021-00218-w (PMC7849121; doi:10.1186/s13722-021-00218-w)
Supplement: Supplementary file 2 — Additional file 2: Appendix S2. Baseline PROUD implementation monitoring information. [file 13722_2021_218_MOESM2_ESM.docx]

**Baseline PROUD implementation monitoring information**

**Please fill this out and send back to the PROUD mail box.**

**Staffing:** For each clinic that will be randomized, please give an estimate of primary care staffing:

| Clinician | Number on staff | %FTE Total |
| --- | --- | --- |
| Providers (MD, DO, NP, PA):   - With buprenorphine waiver - Without buprenorphine waivers |  |  |
| Residents:   - With buprenorphine waivers - Without buprenorphine waivers |  |  |
| RN |  |  |
| LVN/LPN |  |  |
| Medical Assistant (MA) or Health Tech (HT) |  |  |
| Social Worker |  |  |
| Masters level or PhD psychologist |  |  |
| General psychiatrist |  |  |
| Addictions psychiatrist |  |  |
| Peer recovery coaches |  |  |
| Other staff who have patient contact (e.g. front desk staff): |  |  |

**Baseline debrief between Site PI/PM and PROUD Implementation Monitoring Team.**

Below are the questions we will ask at our baseline check-in. Please be prepared to answer the questions for each clinic (separately). We anticipate this will require you to interview your health system stakeholders (e.g. clinic manager and/or chief, medical chief, etc.) of each clinic.

1. **In your opinion** (Site PI/PM), on a scale of 0 to 10, where 0 is poor and 10 is excellent:
   - How would you rate the level of access patients, in each clinic, have to OUD treatment? Why?
   - How would you rate the quality of care each clinic provides for patients with OUD? Why?
   - How would you rate the level of support from clinic staff for providing buprenorphine for OUD in primary care in each of your clinics that will be randomized? Why?
2. **Screening in each of your randomized primary care clinics (separately):**

Is there screening for drug use or opioid use disorder (OUD) in primary care? If yes:

- - When and how often does it take place (e.g. annually, provider discretion, etc.)?
  - How does it occur (e.g. on paper, verbal, online health risk assessment, etc.)? Are these included in the electronic health record?
  - Is screening based on standardized screening questions or a clinical assessment?

1. **Chronic prescription opioid therapy for non-cancer pain in each of your randomized primary care clinics (separately):**

- When patients are on chronic prescription opioids for pain, what policy requirements are currently in place regarding assessment (e.g. behavioral health clinicians or pain specialists, etc.), monitoring (e.g. urine drug screens), or Prescription Drug Monitoring Program (PDMP) use? We are interested in policies from your state, insurers (e.g. Medicaid), the health system, or local clinic.
- Are there any changes in how chronic prescription opioid therapy is managed for chronic non-cancer pain in the clinic?
  - Have there been any changes in the past year or two?
  - Are you anticipating any changes in the next 6 months (e.g. initiatives in health system, local, state, etc.)?

1. **Treatment practices for opioid use disorders (OUD) in each of your randomized primary care clinics (separately):**

- Tell us about the ways that patients learn about OUD treatment options.
- When patients call primary care seeking treatment for an OUD, what typically happens?
- When primary care providers are concerned that a patient *might* have an OUD, what are the providers’ options?
- When patients are *diagnosed* with OUDs in primary care, what are their options?
- Where can patients get buprenorphine treatment? What are the wait times?
- What are the options for methadone treatment for OUDs for patients in each clinic? What are the wait times?
- Is injectable naltrexone offered for alcohol or opioid use disorders? If so, where? Can injectable naltrexone for OUDs be obtained without prior authorization (for most patients with OUD)?

1. Are there any **changes in how OUD treatment is managed that you are anticipating** in your health system in the next 6 months (e.g. initiatives in health system, local, state, etc.)? Please indicate if these vary across the clinics being randomized.
