# Supplementary material for: PRimary Care Opioid Use Disorders treatment (PROUD) trial protocol: a pragmatic, cluster-randomized implementation trial in primary care for opioid use disorder treatment
Source: Addict Sci Clin Pract. 2021 Jan 31;16:9. doi: 10.1186/s13722-021-00218-w (PMC7849121; doi:10.1186/s13722-021-00218-w)
Supplement: Supplementary file 3 — Additional file 3: Appendix S3. Clinic staff survey. [file 13722_2021_218_MOESM3_ESM.docx]

**INSTRUCTIONS**: *Your health system is participating in a new study about opioid addiction treatment in primary care. We are interested in all primary care clinic staff’s opinions about treating opioid addiction in primary care, including what you think about using medications to treat opioid addiction, like buprenorphine (e.g., Suboxone) and naltrexone (e.g., Vivitrol). There are no right or wrong answers. Please select the best response for each question.*

***(1) What is your professional role in this primary care clinic?***

- MD/DO 🡪 Do you have a buprenorphine waiver? 🔾 NO 🔾 YES 🔾 in process of waiver training
- NP/PA 🡪 Do you have a buprenorphine waiver? 🔾 NO 🔾 YES 🔾 in process of waiver training
- Nurse (RN/LPN)
- MA/health tech
- Administrative 🡪 please specify: _____________________________________________
- Other 🡪 please specify: _____________________________________________

***(2) How long have you practiced in this professional role?*** *___ ___ (years) ___ ___ (months)*

***(3) How long have you worked in this primary care clinic?*** *___ ___ (years) ___ ___ (months)*

***(4) People who work in this clinic think providing opioid addiction treatment in primary care is important.***

| Strongly Disagree | Disagree | Neither Disagree nor Agree | Agree | Strongly Agree |
| --- | --- | --- | --- | --- |
| **1** | **2** | **3** | **4** | **5** |

***(5) I welcome opioid addiction treatment in our primary care clinic.***

| Strongly Disagree | Disagree | Neither Disagree nor Agree | Agree | Strongly Agree |
| --- | --- | --- | --- | --- |
| **1** | **2** | **3** | **4** | **5** |

***(6) I think our clinic’s patients would benefit if we offered medications for opioid addiction, like buprenorphine or naltrexone.***

| Strongly Disagree | Disagree | Neither Disagree nor Agree | Agree | Strongly Agree |
| --- | --- | --- | --- | --- |
| **1** | **2** | **3** | **4** | **5** |

***(7) People who work here are motivated to provide treatment for patients with opioid addiction.***

| Strongly Disagree | Disagree | Neither Disagree nor Agree | Agree | Strongly Agree |
| --- | --- | --- | --- | --- |
| **1** | **2** | **3** | **4** | **5** |

***(8) Treating opioid addiction in our clinic would have a positive effect on our operations.***

| Strongly Disagree | Disagree | Neither Disagree nor Agree | Agree | Strongly Agree |
| --- | --- | --- | --- | --- |
| **1** | **2** | **3** | **4** | **5** |

***(9) Our clinic’s leadership is strongly committed to treating opioid addiction with medications, like buprenorphine and naltrexone.***

| Strongly Disagree | Disagree | Neither Disagree nor Agree | Agree | Strongly Agree |
| --- | --- | --- | --- | --- |
| **1** | **2** | **3** | **4** | **5** |

***(10) Treating patients with opioid addiction with medications, like buprenorphine or naltrexone, seems like a good fit for our clinic.***

| Strongly Disagree | Disagree | Neither Disagree nor Agree | Agree | Strongly Agree |
| --- | --- | --- | --- | --- |
| **1** | **2** | **3** | **4** | **5** |

***(11) Treating opioid addiction in our clinic with medications, like buprenorphine or naltrexone, seems doable.***

| Strongly Disagree | Disagree | Neither Disagree nor Agree | Agree | Strongly Agree |
| --- | --- | --- | --- | --- |
| **1** | **2** | **3** | **4** | **5** |

***Thank you for your time!***
