# Supplementary material for: PRimary Care Opioid Use Disorders treatment (PROUD) trial protocol: a pragmatic, cluster-randomized implementation trial in primary care for opioid use disorder treatment
Source: Addict Sci Clin Pract. 2021 Jan 31;16:9. doi: 10.1186/s13722-021-00218-w (PMC7849121; doi:10.1186/s13722-021-00218-w)
Supplement: Supplementary file 4 — Additional file 4: Appendix S4. Quarterly PROUD debrief between Site PI/PM and PROUD Implementation Monitoring Team. [file 13722_2021_218_MOESM4_ESM.docx]

**Quarterly PROUD debrief between SITE PI/PM and PROUD Implementation Monitoring Team**

These questions are for you to use when conducting stakeholder interviews with health system stakeholders (e.g. clinic manager and/or chief, medical chief, etc.) of each clinic. Please be prepared to debrief answers with the PROUD Implementation Monitoring Team and address each question for each clinic (separately).

**Questions for stakeholder interviews:**

We are here to find out what’s changed in your clinic since our last interview. Please tell me about anything that’s changed in your clinic.

Please tell me about general changes regarding:

- Leadership in the health system and in your clinic
- Staffing in your clinic
- Meetings and communication in your clinic
- Quality improvement processes in your clinic
- New patient populations (e.g. from new contracts with Medicaid, the military, larger employers)
- Other organizational changes
- Any recent challenges your clinic is facing

For the following questions, please think about health system policies, guidelines, and practices or new state and local laws, community services and resources, and reimbursement that have changed or are affecting your clinic regarding:

- Standardized screening for substance use
- Treatment of opioid use disorders, including access, cost, and wait times for treatment
  - Buprenorphine
  - Methadone
  - Injectable naltrexone
- Behavioral health treatment (specialty and in primary care)
- Chronic opioid therapy treatment and pain management
- Naloxone use
- The health insurance environment that would affect your patients’ ability to get treatment for opioid use disorders covered or reimbursed?
- If you have a team member addressing opioid use disorders, how has that affected your clinic?
- What are patients’ biggest challenges for getting medication treatment for opioid use disorders?

**Question for Site PI and Project Manager to answer at Implementation Monitoring Team debrief***:*

- What do you think is going well and not going well in the randomized clinics?
